# Increased liver carcinogenesis and enrichment of stem cell properties in livers of Dickkopf 2 (Dkk2) deleted mice

**DOI:** 10.18632/oncotarget.3293

**Published:** 2015-03-16

**Authors:** Thorsten Maass, Jens Marquardt, Ju-Seog Lee, Markus Krupp, Peter Scholz-Kreisel, Carolin Mogler, Peter Schirmacher, Martina Müller, Heiner Westphal, Peter R. Galle, Andreas Teufel

**Affiliations:** ^1^ Department of Internal Medicine I, University of Regensburg, Regensburg, Germany; ^2^ I. Department of Medicine, University Medical Center Mainz, Mainz, Germany; ^3^ Cancer Biology Program, MD Anderson Cancer Center, Houston, TX, USA; ^4^ Department of Informatics, Johannes Gutenberg University Mainz, Mainz, Germany; ^5^ Institute of Pathology, University of Heidelberg, Heidelberg, Germany; ^6^ Laboratory of Mammalian Genes and Development, National Institute of Child Health and Human Development, National Institutes of Health, Bethesda, MD, USA

**Keywords:** transcriptomics profiling, prognostic signature, genetic signature, Dkk2, stem cells

## Abstract

Dkk2 a antagonist of the Wnt/β-catenin-signaling pathway was shown to be silenced in diverse cancers. More recent data indicate that Dkk family members may also possess functions independent of Wnt-signaling during carcinogenesis. The detailed biological function of Dkks and its relevance for liver cancer is unknown. We analyzed the effects of a genetic deletion of Dkk2 (Dkk2^−/−^) in a hepatocarcinogenesis model using DEN/Phenobarbital. Untreated Dkk2^−/−^ animals, showed considerable atypia with variation of hepatocyte size and chromatin density. In livers of Dkk2^−/−^ mice nodule formation was seen at 9 months of age with focal loss of trabecular architecture and atypical hepatocytes and after DEN induction Dkk2^−/−^ mice developed significantly more liver tumors compared to controls. Whole transcriptome analysis of untreated Dkk2^−/−^ liver tissue revealed a Dkk2-dependent genetic network involving Wnt/β-Catenin but also multiple additional oncogenic factors, such as e.g. Pdgf-b, Gdf-15 and Hnf4a. Dkk2^−/−^ tumor cells showed a significant deregulation of stemness genes associated with enhanced colony forming properties. Integration of the Dkk2^−/−^ signature into human data was strongly associated with patients survival. Dkk2 deletion results in alterations of liver morphology leading to an increased frequency of liver cancer. The associated genetic changes included factors not primarily related to Wnt/β-Catenin-signaling and correlated with the clinical outcome of HCC-patients.

## INTRODUCTION

Hepatocellular carcinoma (HCC) ranks among the most common cancers and is the third leading cause of cancer-related death worldwide. Each year approximately 500 000 patients are diagnosed with the disease. However, due to late detection most patients are not eligible for curative treatment options such as surgical resection, radio-frequency ablation or liver transplantation. Simultaneously, palliative treatment options are also very limited with sorafenib currently being the only approved substance for systemic therapy and a survival benefit of only 3 months [1]. Therefore, improving the understanding of underlying molecular mechanisms, genetic pathways and, in particular, a detailed in depth analysis of genetic drivers of hepatocarcinogenesis is critical to foster the progress in therapy for HCC patients [2–4].

Changes in gene expression or mutations of members of the Wnt signaling pathway are relevant and early events in hepatocarcinogenesis. Most recent next generation sequencing analyses confirmed that genetic alterations of p53 and β-catenin are among the most prevalent changes in HCC [5, 6]. However, alterations of other members of the WNT signaling pathway constitute alternative ways of β-catenin activation. Finally, in-depth analyses of the downstream signaling are urgently needed to understand the complex molecular interactions leading to WNT/β-catenin driven liver cancer development [7–10].

As part of the Wnt signaling pathway the Dickkopf (Dkk) gene family members act as antagonistic regulators of the Wnt signaling pathway and the Wnt co-receptors LRP5/6 class by forming a complex with Wnt and Frizzled proteins [11–13]. The Dkk family consists of four main members (Dkk1-Dkk4), which contain two distinct cysteine-rich domains [14]. DKK1 is typically silenced in colon cancer by hypermethylation, and its methylation status correlates with progression in advanced tumor stages [15]. DKK3 has also been reported to act as a tumor suppressor in various malignancies, such as breast, pancreatic, cervical, bladder, prostate, renal, and non small cell lung cancer, as well as leukemia [16, 17].

DKK2 is thought to directly inhibit binding of Wnt to the LRP5/LRP6 co-receptors of FZD. In melanoma and gastrointestinal cancers, DKK2 expression is markedly decreased [18, 19]; however, the details of Dkk2 function were not well investigated.

In recent years several Wnt/β-catenin independent functions were postulated for DKK1 and DKK2. For example, Dkk1 overexpression in the mesothelioma cell line H28 caused apoptosis and growth inhibition despite a homozygous deletion of β-catenin in these cells [20]. Furthermore, DKK2 was demonstrated to influence the differentiation of bone precursor cells over a BMP-p53-Osterix-ALP axis and to stimulate the expression of the NF-kappaB ligand (RANKL) [21].

In summary, the Dkk family is highly important for carcinogenesis in various tissues. While part of its action is clearly exerted through regulation of the Wnt signaling pathway, diverse other signaling pathways may be relevant effectors of Dkk signaling. In the present study we investigated the role of Dkk2 in hepatocarcinogenesis using a model of Dkk2 deficient mice and subsequently applied advanced bioinformatics profiling. As a result we could attribute a key role for Dkk2 in preventing liver cancer development by interacting with WNT/β-catenin as well as other key oncogenes. Furthermore, induction of stemness-associated genes in liver tumors of Dkk2 deficient animals indicates a potential disruption of Dkk2 signaling in liver stem/progenitor cells during the course of carcinogenesis. Consistently, Dkk2 dependent gene networks were associated with poor prognosis of HCC patients in 2 independent cohorts of 252 HCC.

## RESULTS

### Dkk2^−/−^ deletion leads to an enhanced development of liver cancer

**Figure 1:**
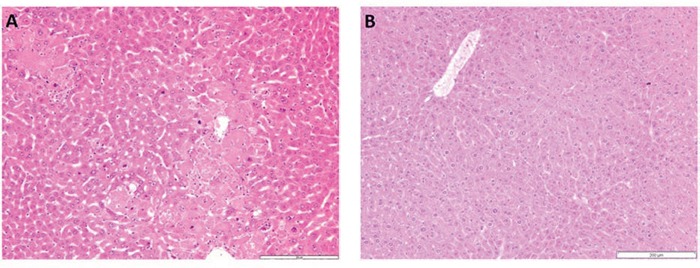
**A. H&E staining of Dkk2^−/−^ liver tissue. Dkk2 deleted animals showed moderate to focally stronger variation of hepatocyte size with special regard to variable nuclear size and chromatin density. B.** Corresponding wild type liver tissue with regular trabecular architecture and uniform hepatocytes.

**Figure 2:**
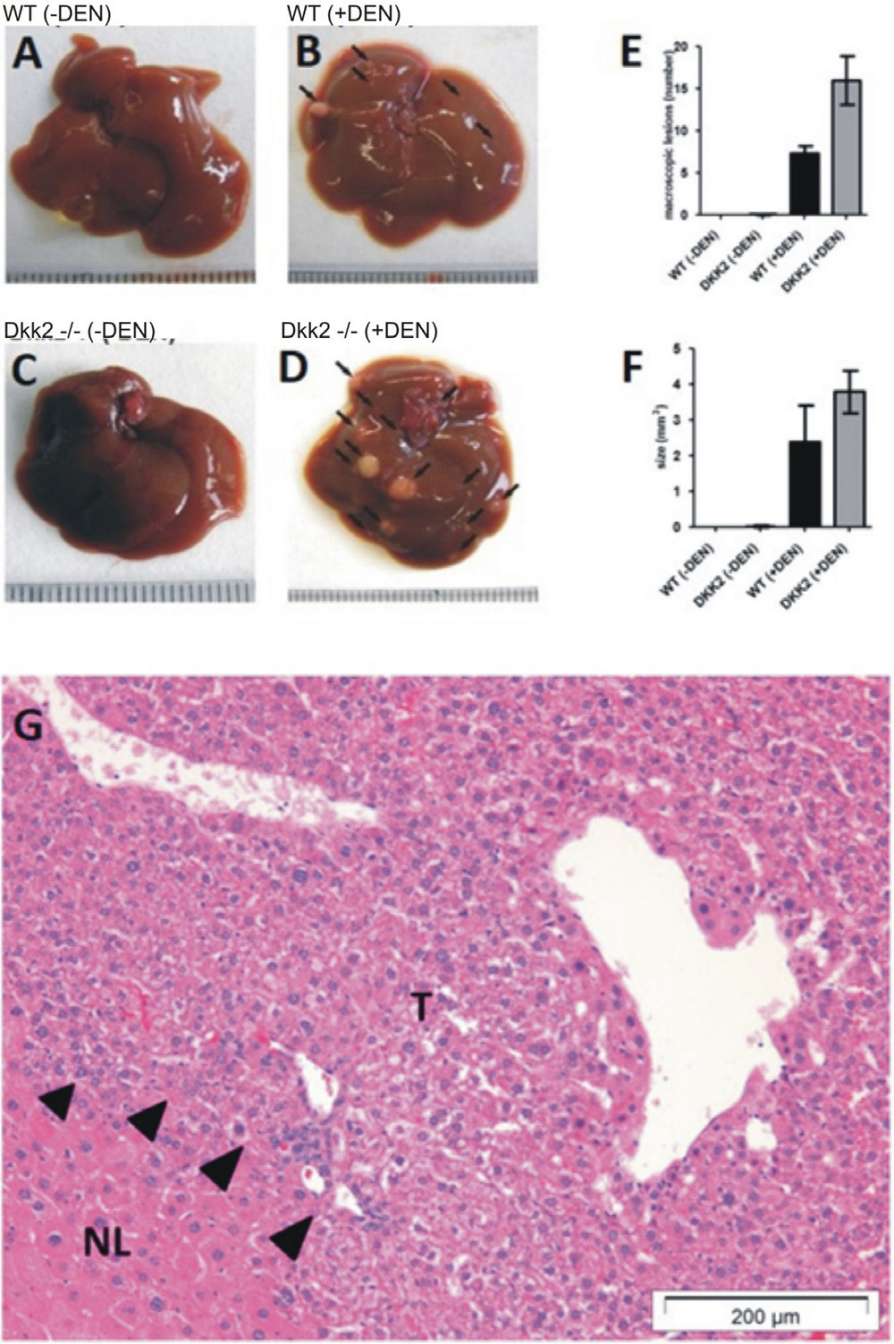
**A-D. Tumor development in Dkk2^−/−^ knock out and wild type (WT) animals demonstrated a significantly increased tumor development in diethylnitrosamine (DEN) treated Dkk2^−/−^ compared to WT animals.** Untreated animals did not shown any spontaneous tumor development at 9 months age. **E.** Number of macroscopically visible lesions. **F.** Size of macroscopically visible lesions. **G.** Histological confirmation of tumor development. H&E stained liver section showing normal and cancerous (arrows) tissue. Data are presented as means ± SEM.

Liver cancer development was induced using the DEN/phenobarbital tumor model (Fig. 2G). Livers of Dkk2^−/−^ and WT mice were sacrificed at 9 months of age to investigate morphological changes during tumor development. At 9 months livers of WT and Dkk2^−/−^ animals did not show any significant differences with regard to liver size, weight, or macroscopic appearance. Histologically, liver architecture with portal tracts, central veins, trabecular arrayed hepatocytes and sinusoids was identical in Dkk2^−/−^ and WT mice. Nine months after DEN induction WT animals showed mild perisinusoidal fibrosis and few atypical hepatocytes. In contrast, Dkk2 deficient animals showed moderate hepatocellular atypia with anisocytosis–caryosis and nuclear hyperchromasia and developed hepatocellular carcinomas with loss of trabecular architecture resembling typical DEN induced liver tumors (Fig. 1). In addition, mild peritumoral inflammation and fatty change was observable.

Macroscopically, numbers of tumor nodules detectable on the liver surface were significantly increased in Dkk2^−/−^ compared to WT mice (Fig. 2A-D). Besides the higher incidence of tumors Dkk2^−/−^ mice exhibited a highly significantly increased volume of the tumor nodules in comparison to WT mice (Fig. 2F). On average the Dkk2^−/−^ livers contained average 16 tumor nodules (*n* = 11) whereas the age matched, WT littermates contained average only 7.4 tumor nodules (*n* = 10) (Fig. 2E). Consistent with the accelerated tumor growth a significantly increased proliferation rate could be detected in untreated, non-tumorous Dkk2^−/−^ deleted livers.

### Global transcriptome analyses of Dkk2^−/−^ livers

To dissect in more detail the underlying molecular mechanisms involved in an increased liver cancer development of Dkk2^−/−^ animals, we performed microarray gene expression analysis of untreated livers from 4 Dkk2^−/−^ and 4 WT animals to identify differentially regulated genes at 9 months of age. A total of 760 differentially expressed genes separated Dkk2^−/−^ from WT livers as demonstrated by unsupervised clustering analyses and principal component analysis. Many of the differentially regulated genes were involved in inflammation and immune response, cell adhesion, as well as apoptosis (Fig. S1A).

Ingenuity pathway analysis of the Dkk2^−/−^ gene expression signature identified a large number of overlapping sub-networks indicating a tight regulatory interaction of the Dkk2 dependent signaling pathways. However, assembling the complete genetic network of potentially interacting genes, we depicted an 825 nodes containing Dkk2^−/−^ modulated network as shown in Fig. S1. Among the nodes connecting the overlapping small functional network were several carcinogenesis associated genes such as Pdgf-b, Gdf-15, Hnf4a, Cd83, and Orm1.

**Figure 3:**
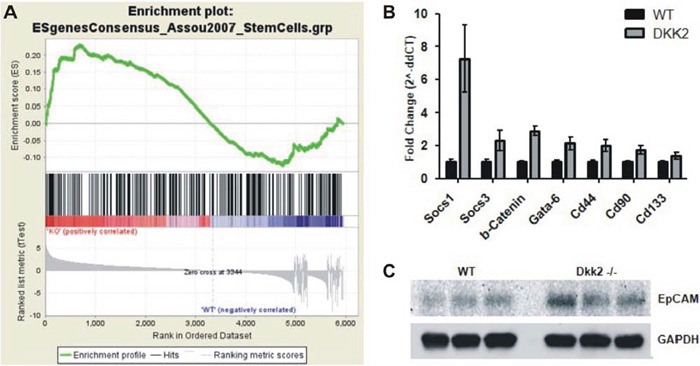
**A. Gene Set Enrichment Analyses (GSEA) of the Dkk2 gene expression signature.** Stem cell associated signature “ESgenesConsensus_assou2007_StemCells” showed a trend to enrichment; *p*-value = 0.057. **B.** Analysis for differential expression by RT-PCR demonstrated a significant over expression of stem cell related genes SOCS1, SOCS3, GATA-6, CD44, CD90, and CD133. **C.** Validation of differential EpCAM protein expression by western blot. Data are presented as means ± SEM.

In order to further pinpoint disturbed biofunctions due to Dkk2 deletion in the liver we also applied Gene Set Enrichment Analyses (GSEA) to the Dkk2 gene expression signature. Consistent with the key role of Dkk2 for Wnt/β-Catenin signaling an enrichment of several signatures such as “MORF_WNT1” (*p*-value 0.009) could be detected. Besides this expected enrichment we also identified enrichment of stem cell and stem cell associated signatures such as “CAR_IGFBP1” (*p*-value 0.035) and (ESgenesConsensus_assou2007_StemCells” (FWER *p*-value = 0.057) as well as prognostic signatures in human liver tumors such as “CAIRO_HEPATOBLASTOMA_POOR_SURVIVAL” (*p*-value = 0.00) or “WANG_RECURRENT_LIVER_CANCER_UP” (*p* = 0.00) for primary and recurrent liver cancer (Fig. 3A).

Selected targets of our microarray results were confirmed in independent liver samples by means of RT-PCR, overall showing a high concordance. Consistently, the top three upregulated genes of the array analysis, Socs-1 (7, 25-fold, *p* = 0, 001), Socs-3 (2, 31-fold, *p* = 0, 014), and Grb10 (4, 92-fold, *p* = 0, 015), as well as the top two downregulated genes Il1b (0, 58-fold, *p* = 0, 024) and Prkca (0, 46-fold, *p* = 0, 001) were independently validated. Furthermore, markers associated with stemness cells such as Epcam, Gata-6, Cd44, and Cd90 were upregulated in Dkk2 deficient livers (Epcam 3.2-fold, *p* = 0.002; Gata-6 2.14-fold, *p* = 0.003; Cd44 1.98-fold; Cd90 1.74-fold, *p* = 0.001). Cd133 showed a trend towards upregulation but closely missed statistical significance (1.38 fold regulation. *p* = 0.056) (Fig. 3B and 3C).

### Colony forming assay

**Figure 4:**
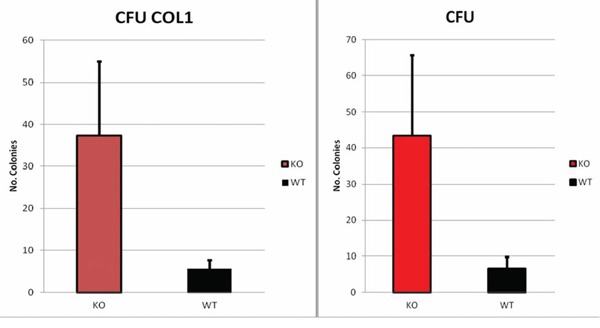
**DKK2^−/−^ cells showed a significant increase in colony forming unit on both collagen coated -CFU COL1- (37 Dkk2^−/−^ and 5 WT-colonies, *p* = 0.03) and normal -CFU- dishes (43 colonies from Dkk2^−/−^ vs. 6 colonies from WT, *p* = 0.04).** Data are presented as means ± SEM.

Colony forming assays of cells isolated from Dkk2^−/−^ and WT tumors at 9 months of age demonstrated that Dkk2^−/−^ cells possess a superior colony formation on both normal (43 colonies from Dkk2^−/−^ vs. 6 colonies from WT, *p* = 0.04) and collagen coated dishes (37 Dkk2^−/−^ and 5 WT-colonies, *p* = 0.03) (Fig. 4).

### Translation of Dkk2^−/−^ dependent networks into human cancer biology

**Figure 5:**
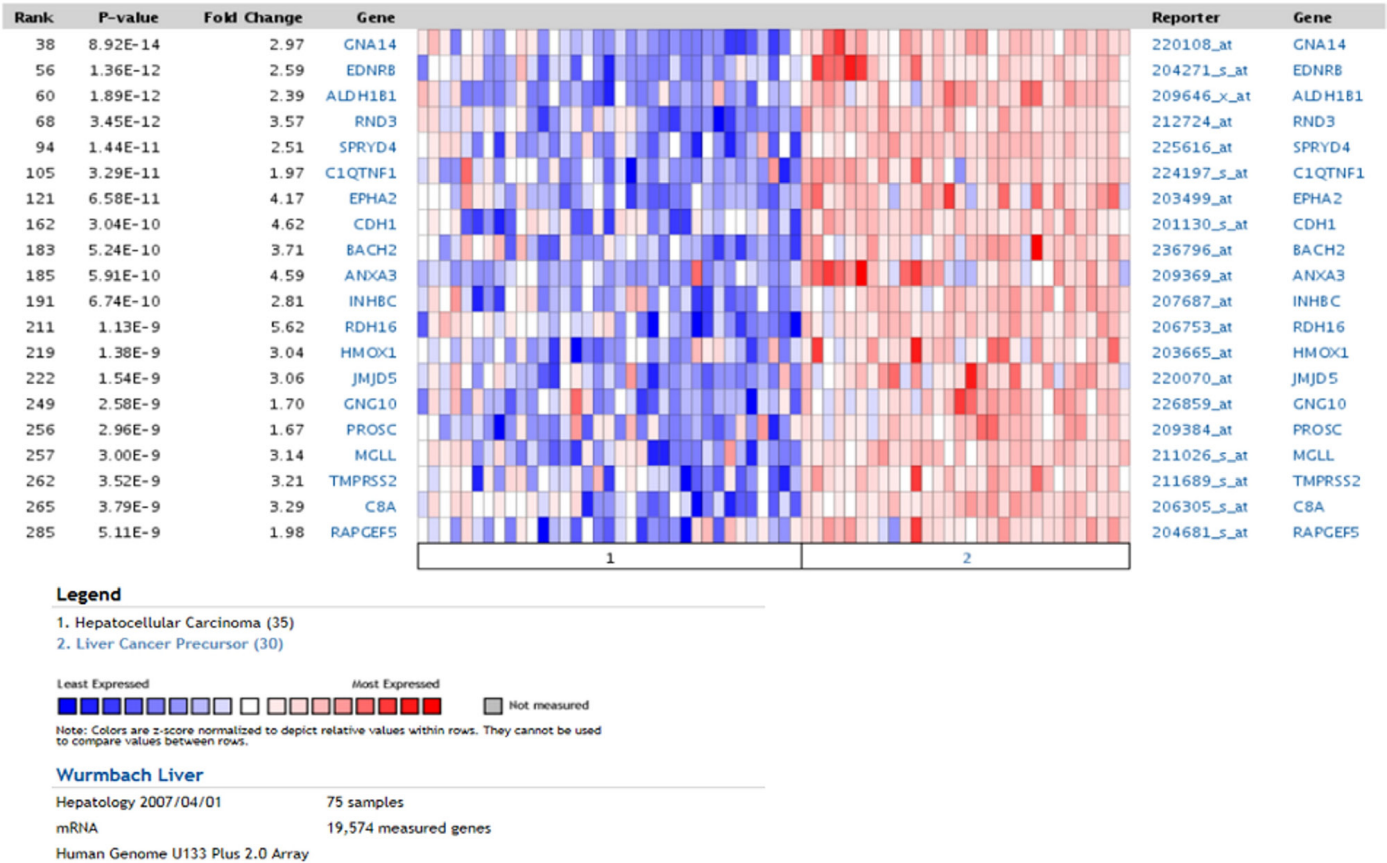
**Oncomine analysis using the Dkk2^−/−^ genetic signature.** Comparing HCC and liver cancer precursors demonstrated a clear distinction between undifferentiated precursors and well differentiated HCC samples.

In order to translate the observed findings to human cancer biology we integrated our signature of differentially regulated genes in dependence of Dkk2 knock out with authentic human tumors. We therefore investigated the expression of these genes in correlation to human tumor development and prognosis. An initial search of the cancer microarray database Oncomine [22] revealed a highly significantly correlated overlap of 100 genes of our Dkk2 network in a HCC data set previously published by Wurmbach et al. [23]. These overlapping gene profiles were furthermore significantly distinct between liver cancer precursors and HCC (*p* = 4.75E-9) (Fig. 5).

**Figure 6:**
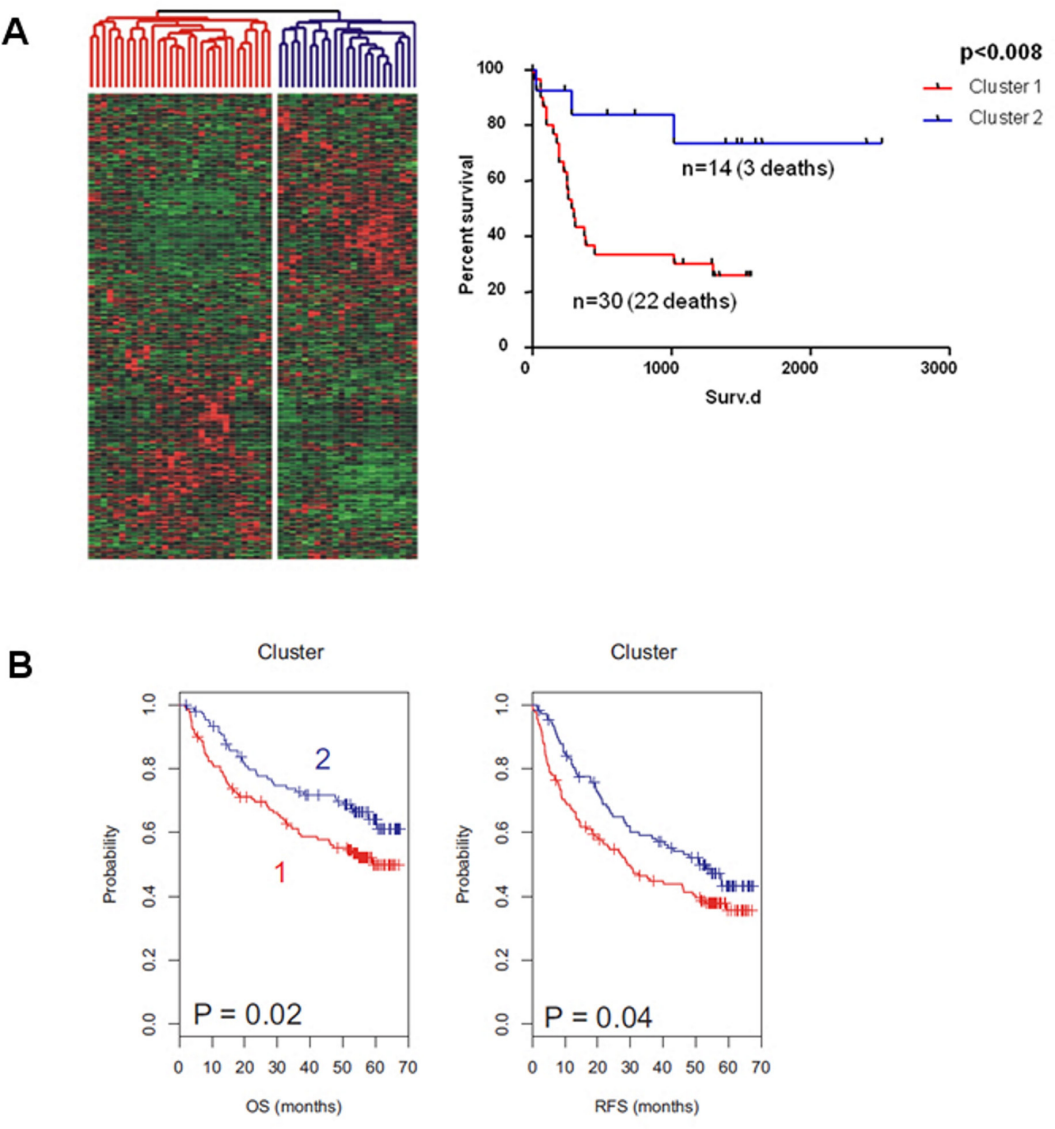
**Unsupervised Clustering of an initial cohort of A. 53 patients with HCC and B. a validation cohort of 199 patients with HCC.** Subsequent Kaplan-Meier-Analysis of the resulting two subgroups demonstrated significantly differing survival of these subgroups in both patient cohorts.

We therefore investigated our Dkk2^−/−^ signature in microarray data from 53 human HCC [24, 25]. Unsupervised clustering on the basis of the Dkk2^−/−^ dependent network genes and subsequent Kaplan Meier analysis of the two resulting groups of patients demonstrated a highly significant association of these genes to overall survival (*p* = 0.008) (Fig. 6A).

This prognostic value was furthermore validated in a second independent cohort of 199 HCC patients (*p* = 0.02, overall survival) [26]. Both, overall survival (*p* = 0.02) and recurrence free survival (*p* = 0.04) differed significantly between these subgroups solely defined by a Dkk2^−/−^ dependent genetic signature (Fig. 6B).

## DISCUSSION

Dkk2 plays a key role in various human tissues and Dkk2 expression is known to be disrupted in multiple cancers including renal cancer, skin cancer, and different gastrointestinal cancers. In many of these cancers *DKK2* was down regulated due to promoter methylation [15, 27, 28]. However, there was no convincing *in vivo* proof that down regulation of Dkk2 in fact modulated genetic changes in and significantly contributes to carcinogenesis. Furthermore, the role of Dkk2 for liver cancer development was completely unknown. Our work now provides definite *in vivo* evidence that genetic loss of Dkk2 contributes to hepatocarcinogenesis and prognosis of patients with liver cancer.

### Carcinogenesis/network biology

DEN treatment of Dkk2^−/−^ KO mice resulted in a significantly increased liver cancer development compared to DEN treated WT mice. In order to identify the molecular mechanisms underlying this increased hepatocarcinogenesis we performed a microarray analysis of the untreated Dkk2^−/−^ tissue compared to WT tissue combined with subsequent advanced bioinformatics characterization. These analyses resulted in the identification of a large network of differentially regulated genes.

### Characterization of a large, differentially regulated genetic network modulated by loss of Dkk2

Dkk2 loss resulted not only in a Wnt/β-catenin activation but also in multiple expression changes of genes and genetic pathways outside Wnt/β-catenin signaling. We confirmed central nodes of this network independently in additional tissue samples by means of RT-PCR. These genes were closely linked to liver cancer before and thus a) demonstrated the close linkage of Dkk2 loss dependent genetic changes to liver cancer b) a functional impact of Dkk2 loss on many diverse genes and genetic networks. We and others have previously demonstrated that overexpression of Pdgf-b is critical to hepatocarcinogenesis [29]. Hepatocyte nuclear factor-4α (Hnf4α), known as the master regulator of hepatocyte differentiation, was also demonstrated to be closely linked to hepatocarcinogenesis since an extensive microarray analysis on Hnf4a^−/−^ mice suggested that its loss may directly contribute to hepatocellular carcinoma [30, 31]. Furthermore, an HNF4α-miRNA inflammatory feedback loop was shown to regulate hepatocellular oncogenesis [32]. Orosomucoids (ORMs) were discussed – although not in HCC- to function as a blocking factor protecting tumor cells against immunological attack, thereby contributing to the ‘immune escape’ of the tumor, independent of Wnt signaling [33]. Finally, growth/differentiation factor-15 (GDF-15), a TGF-beta family member, was expressed in response to liver injury and carcinogen exposure and was highly associated with stage, size, metastasis as well as inhibition of tumor growth and increased tumor invasiveness in gastrointestinal cancers. However, genetic ablation of GDF-15 alone had no apparent effect on HCC tumor formation rate, growth rate, or invasiveness; thus GDF-15 may rather exert its effects in context of pro-carcinogenetic networks [34].

In summary, these genes have been closely linked to liver cancer before and validation of their differential expression furthermore clearly demonstrated that Dkk2 loss results in a broad interference with multiple genetic pathways not primarily linked to Wnt/β-catenin signaling ultimately leading to HCC development.

### Enriched stem cell markers in Dkk2^−/−^ deleted livers

Stem cell biology has become widely studied in recent years with respect to tissue regeneration but also cancer development. In contrast to other cancers, the existence of cancer stem cells in HCC has still not been proven. However, several recent publications suggest that cells with stem cell properties and markers contribute to hepatocarcinogenesis. As an approach towards investigating a potential role of stem cells in HCC several groups have analyzed stem cell signatures identified in normal tissue or other cancers in HCC and were able to correlate the expression of these genes with survival of patients, tumor progression, and response to treatment. One of the pioneering publications was published by Lee et al. [35], describing genetically distinct subtypes of HCC. Patient subgroups that share gene expression with stem cell signatures had a worse outcome and shorter survival. This result is in agreement with findings from other tumors in which CSC had a higher proliferative potential and enhanced anti-apoptotic properties resulting in a higher tumorigenic potential [36]. The enrichment of stem cell or stem cells related signatures in Dkk2^−/−^ livers prompted us to further search for stem cell related changes of gene expression. Western blot or RT-PCR confirmed upregulation of several known stemness markers, such as EpCAM, CD133, CD90, and CD44 in Dkk2^−/−^ animals. The EpCAM positive subpopulation EpCAM+ from HCC cells are thought to possess stem cell properties, as they exhibited a greater proliferative capacity *in vitro* and *in vivo* compared to EpCAM− cells [37]. Furthermore, the hepatic stem cell (HSC) marker CD133 has been suggested as a potential marker of HCC CSC as CD133 positive cells possess several characteristics previously attributed to CSC in other tumors such as high tumorigenic capacity, higher chemoresistance but also a significantly higher expression of stem cell associated genes. Understanding the characteristics and function of CD133^+^ liver CSCs has also shed light on HCC management and treatment, including the implications for prognosis, prediction and treatment resistance [38]. Recently, CD90^+^CD44^+^ cells were reported to engraft in the livers of severe combined immunodeficient mice and the concomitant expression of CD44 (CD90^+^CD44^+^ liver CSCs) seems to correlate with an aggressive growth pattern of HCC.

In our Dkk2^−/−^ livers all of the above mentioned stem cell associated factors were demonstrated to undergo severe deregulation compared to livers from wild-type animals, indicating a close link between Dkk2 ablation in liver tissue and stem cell biology.

### Prediction of survival of patients with hepatocellular carcinoma

Another key finding of our study is the prognostic implication of the identified Dkk2 signature for human liver cancer. Over the past decade, more than 20 clinically relevant gene expression signatures have been generated in HCC [4]. However, only very few were validated in independent patient cohorts and thus, the robustness of these signatures remains to be demonstrated. We have therefore validated our signature which was identified in 53 patients with HCC in a second independent, cohort of 199 HCC patients. Since our signature was demonstrated to characterize significant differences in both overall and progression free survival and was validated in two independent cohorts it may be considered to rank among the more robust signatures developed.

Besides its pure role in prognosis, this link to patient's outcome suggests a high relevance of the genes of this signature for the relevant biological changes related to liver cancer development.

## MATERIALS AND METHODS

### Dkk2-knockout-model

For all experiments a constitutive Dkk2^−/−^-knockout mouse on C57Bl/6-backgound was used [39]. Age-matched wild-type (wt) littermates were used as controls. All experiments were performed in accordance with the governmental and institutional guidelines and with written approval of the state animal care commission.

### Induction of carcinogenesis

To induce liver carcinogenesis a single diethylnitrosamine (DEN) i.p. injection (0.05 mg per animal) was performed at day 7 post-partum. Promotion of carcinogenesis was achieved by continuously adding phenobarbital (0.05% w/v) to the drinking water. Mice were sacrificed after 9 months and analyzed for liver cancer development [40].

### Initial liver analysis and immunohistochemistry

All livers had been characterized by their size, weight, number, and macroscopic size of tumor nodules. For further analyses of the liver structure samples were fixed in formalin, embedded in paraffin, and stained with hematoxylin and eosin (H&E). Immunohistochemistry was performed on frozen sections (6 μm thick). Ki-67 staining was performed on acetone-fixed sections by using an rat-anti-mouse Ki-67 (1:50, DakoCytomation, Glostrup, Denmark) as primary antibody. Signal detection was performed using ‘Vectastain ABC Kit’ (Vector Laboratories, Burlingame, USA) and ‘Fuchsin Substrate-Chromogen System’ (DakoCytomation). As a measurement of proliferation the total number of Ki-67-positive and −negative cells in the liver tissue was counted. In total, more than 5000 cells were counted on liver sections from both Dkk^−/−^ and WT mice.

### Microarray analysis of RNA from untreated Dkk2^−/−^ and WT liver tissue

Microarray analysis was performed by atlas biolabs on an Illumina mouse ref-8 beadchip (Illumina, San Diego, CA, USA). Data was analyzed using BRB ArrayTools V4.2.1 software package 3 (Biometric Research Branch, National Cancer Institute, USA). The data was normalized using quantile normalization and differentially expressed genes were identified at a nominal *p*-value ≤ 0.01 using a univariate test with 10000 permutations. Unsupervised cluster analysis was performed using Cluster and TreeView 2 program. (http://www.genealogyreviews.co.uk/fhmMil10treeview.htm). Functional classification and network analysis were performed using Ingenuity Pathway Analysis tool (Ingenuity Systems, Redwood City, CA, USA, http://www.ingenuity.com), GSEA (http://www.broadinstitute.org/gsea/index.jsp) and the GeneGo microarray tool (http://portal.genego.com).

### RealTime-PCR

Total RNA was isolated using ‘Tris Reagent’ (Sigma–Aldrich, Taufkirchen, Germany) and cDNA was applied from 0.5 μg total RNA with oligo-dt-primers by using the ‘First Strand cDNA Synthesis Kit’ for RT-PCR (AMV), (Roche, Mannheim, Germany) both according to the manufacturer's instructions.

Specific mRNA transcripts were quantified with a LightCycler (Roche) by using ‘LightCycler Fast Start DNA Master SYBR Green I’ (Roche) and the following primers: GAPDH (GenBank Acc. No. NM_001303; for: GGCATTGCTCTCAATGACAA; rev: TGTGAGGGAGATGCTCAGTG) and rS6 (GenBank Acc. No. NM_009096; for: GTCCGCCAGTATGTTGTCAG; rev: GTTGCAGGACACGAGGAGTA) as housekeeping-genes; c-myc (GenBank Acc. No. NM_010849; for: TCCTGTACCTCGTCCGATTC; rev: GGTTTGCCTCTTCTCCACAG), Cyclin D1 (GenBank Acc. No. NM_007631; for: CACAACGCACTTTCTTTCCA; rev: ACCAGCCTCTTCCTCCACTT), p21 (GenBank Acc. No. NM_007669; for: TCTTGCACTCTGGTGTCTGA; rev: TTCAGGGTTTTCTCTTGCAG), and EpCAM (Tacstd1, GenBank Acc. No. NM_008532, for: 5ʹ-TGTGGTGGTGTCATTAGCAG-3ʹ, rev: 5ʹ-GGATCTCACCCATCTCCTTT-3ʹ) according to the manufacturer's instructions. Determination of gene expression was performed by using the LightCycler software package. Relative gene expression was given as x-fold expression of the used housekeeping gene GAPDH.

### Statistical analysis

Data is presented as mean ± standard error of mean as indicated. For comparison of experimental groups, the nonparametric Mann-Whitney-*U*-test or Student's *t*-test were applied. *P* values < 0.05 were considered statistically significant and *p* < 0.01 was considered to be highly significant.

### Tumor processing

Tumors were resected in toto and washed 3x with ice-cold PBS containing 1% penicillin/streptomycin. Tumors were minced down to < 1 mm pieces, washed with HBSS without Mg^2+^/Ca^2+^ containing 1 M HEPES and 0.2 M EGTA and incubated in a shaking water bath at 37^°^C for 15 min. After discarding the supernatant, cell fragments were incubated in Collagenase IV (GIBCO) for 20 min at 37^°^C followed by the consecutive digestion with trypsin for 10–15 min. The single cell suspension was diluted in a regular medium containing 10% FBS, centrifuged at 1000 rpm for 5 min and plated in 10 cm culture dishes.

### Colony formation

10^3^ Dkk2^−/−^-and WT cells were plated on normal 6-well or collagen 1 coated plates. All experiments were performed in triplicates. Colony formation was monitored for 14 days and colony formation unit (number of colonies/seeded cells) was calculated.

## SUPPLEMENTARY FIGURE


