# Supplementary material for: Increased liver carcinogenesis and enrichment of stem cell properties in livers of Dickkopf 2 (Dkk2) deleted mice
Source: Oncotarget. 2015 Mar 16;7(20):28903–13. doi: 10.18632/oncotarget.3293 (PMC5045365; doi:10.18632/oncotarget.3293)
Supplement: Supplementary file 1 [file oncotarget-07-28903-s001.pdf]

Increased liver carcinogenesis and enrichment of stem cell properties in livers of Dickkopf 2 (Dkk2) deleted mice

SUPPLEMENTARY FIGURE

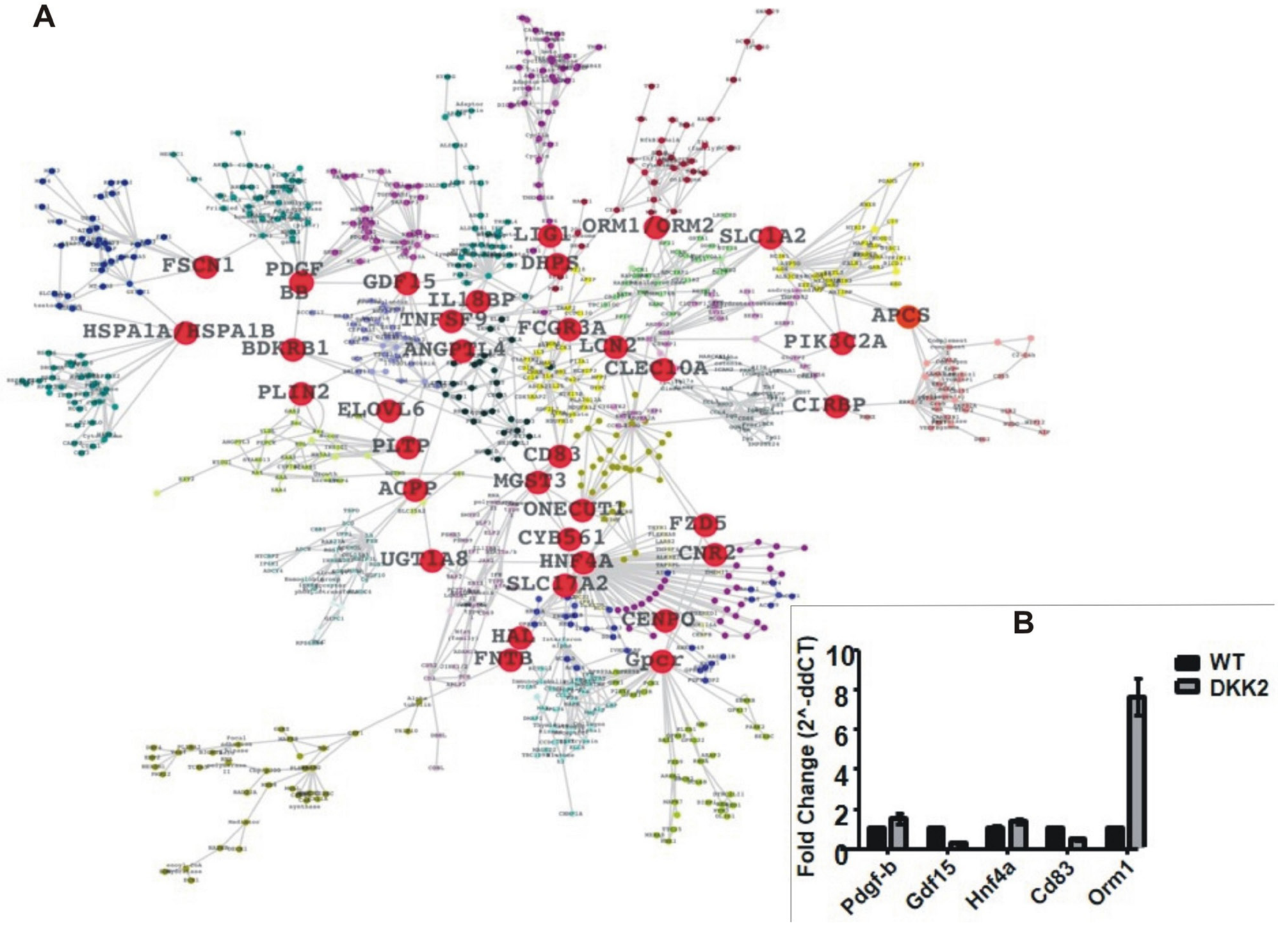

Supplementary Figure S1: A. Merged ingenuity networks showing a high interaction and overlap between networks. All genes of a specific subnetwork defined by biological functions are labeled in the same color. Genes overlapping between multiple networks are labeled in red. **B.** Validation of network nodes by means of RT-PCR. PDGF-B, HNF4A, and ORM1 were upregulated and GDF15 and CD83 were down regulated in livers of Dkk2 −/− compared to livers of WT animals. Data are presented as means ± SEM.
